# The worse the better? Quantile treatment effects of a conditional cash transfer programme on mental health

**DOI:** 10.1093/heapol/czaa079

**Published:** 2020-09-03

**Authors:** Julius Ohrnberger, Eleonora Fichera, Matt Sutton, Laura Anselmi

**Affiliations:** 1 School of Public Health, Department of Infectious Disease Epidemiology, Imperial College London, Medical School Building, St Mary’s Campus, Norfolk Place, W2 1PG, London, UK; 2 Department of Economics, University of Bath, Claverton Down, Bath BA2 7JP, Bath, UK; 3 Institute for Health Policy and Organisation, University of Manchester, Booth Street West, M15 6PB, Manchester, UK

**Keywords:** Malawi, mental health, conditional cash transfer, randomized controlled trial, quantile treatment effect, panel data, low-income countries

## Abstract

Poor mental health is a pressing global health problem, with high prevalence among poor populations from low-income countries. Existing studies of conditional cash transfer (CCT) effects on mental health have found positive effects. However, there is a gap in the literature on population-wide effects of cash transfers on mental health and if and how these vary by the severity of mental illness. We use the Malawian Longitudinal Study of Family and Health containing 790 adult participants in the Malawi Incentive Programme, a year-long randomized controlled trial. We estimate average and distributional quantile treatment effects and we examine how these effects vary by gender, HIV status and usage of the cash transfer. We find that the cash transfer improves mental health on average by 0.1 of a standard deviation. The effect varies strongly along the mental health distribution, with a positive effect for individuals with worst mental health of about four times the size of the average effect. These improvements in mental health are associated with increases in consumption expenditures and expenditures related to economic productivity. Our results show that CCTs can improve adult mental health for the poor living in low-income countries, particularly those with the worst mental health.

**Key Messages**

- First paper that analyses conditional cash transfer (CCT) effects on mental health for the general population and along the mental health distribution.
- CCT improves mental health on average by 0.1 standard deviations with no differences by gender.
- Effects vary along the mental health distribution with strongest effects for those with worst mental health, four times the size than the average effect.
- Improvements in mental health associated with more capabilities to consume and spend on household productivity.

## Introduction

The World Health Organization (WHO) predicts that depression will be the single main contributor to years lived with disability by 2030 (World Health Organization, 2013). This makes poor mental health a pressing global health problem. Populations of low- and middle-income countries (LMICs) are disproportionately negatively affected by mental health problems. About 80% of the world population lives in LMICs, however, <20% of global mental health resources are available in LMICs (Sweetland *et al.*, 2014). Also, research has found a strong negative relationship between income poverty and good mental health (Lund *et al.*, 2010; Hanandita and Tampubolon, 2014) which further exacerbates the burden of mental health in LMICs. Sub-Saharan African LMICs are of particular importance in this context as they house about 415 million or 60% of the poor population globally in 2015 (World Bank, 2018a).

A number of policies have been adopted to tackle poverty, with unconditional cash transfer (UCT) programmes and conditional cash transfer (CCT) programmes being the most popular interventions (Fernald and Gunnar, 2009; Ozer *et al.*, 2009; Paxson and Schady, 2010; Baird *et al.*, 2011; Fernald and Hidrobo, 2011; Ozer *et al.*, 2011; Plagerson *et al.*, 2011; Baird *et al.*, 2013; Eyal and Burns, 2015; Haushofer and Shapiro, 2016; Kilburn *et al.*, 2016, 2019; Angeles *et al.*, 2019). Few studies on populations from low-income countries have analysed the impact of CCT programmes on mental health. The existing studies have found that CCTs reduce stress-levels, psychosocial distress and depressive symptoms, and improve psychosocial well-being (Fernald and Gunnar, 2009; Ozer *et al.*, 2009, 2011; Baird *et al.*, 2013; Kilburn *et al.*, 2019) using population sub-samples of adolescents, children and mothers. None of these studies has either analysed the effects on the wider adult population or tested if the CCT effects are heterogenous in the severity of mental health and more specifically depressive symptoms.

However, understanding both is important for policymakers aiming at sustainably improve mental health in LMICs (Lund *et al.*, 2011; Votruba *et al.*, 2014). While average treatment effects are informative for health efficiency, ignoring heterogeneity may lead to over- or underestimation of the effect for those at worst mental health and at worst rule out programme benefits or even worsen equity in health outcomes. Understanding heterogenous effects in mental health will help identifying differential responses and therefore inform policymakers about which population would benefit most from CCT in terms of mental health and whether those who may need the most would actually benefit (Lund *et al.*, 2011).

Previous research shows that effects of other treatment interventions on mental health differ significantly along the mental health distribution, often with strongest effects for individuals with worst mental health. Stillman *et al.* (2009) analysed the effect of New Zealand’s migration lottery (comparable to the US green-card lottery) on mental health of adults moving from Tonga to New Zealand. The authors found stronger treatment effects for those individuals with worse mental health, compared with those individuals with better mental health. Banerjee *et al.* (2015) analysed a multi-faceted asset-promotion intervention implemented in six LMICs. They found significant positive treatment effects on mental health in the lower and middle part of the mental health distribution, but none for individuals in good to best mental health. The two studies clearly indicate the importance to look beyond mean effects and support the motivation of our analyses.

We aim to fill these gaps in the literature by estimating the average and heterogeneous quantile treatment effects (QTEs) of a CCT on the mental health of the adult population (age 16+) from a low-income country. We use the Malawi Incentive Programme (MIP), a randomized controlled trial (RCT) of a programme that offered cash transfers conditional on maintaining HIV-free status for at least a year. We focus on Malawi due to the high prevalence of mental illnesses in the population. Surveys report that about 30% of the population seeking primary care in Malawi report to have a mental health condition and 19% report to have unipolar depression (Kauye *et al.*, 2014; Udedi, 2014). Our choice of Malawi is further motivated by the high prevalence of poverty in the country with a headcount poverty rate of about 50% (Sub-Saharan Africa 41%) (World Health Organization, 2015; Beegle *et al.*, 2016; World Bank, 2018a,b).

We estimate the average effect and the QTE of this CCT on mental health measured by the SF12 mental health scale which is a general measure of mental health and has been shown to be a good screening tool for mild to moderate common mental health disorders such as psychological distress, depression and anxiety (Vilagut *et al.*, 2013; Ohrnberger *et al.*, 2020). We use two waves (2006 and 2008) of the Malawian Longitudinal Study of Family and Health (MLSFH) conducted shortly before and after the intervention took place. We also test heterogeneity of the effect in mental health by the usage of the cash transfer, and by HIV status and gender of the individual.

### Study setting Malawi

Malawi is a landlocked country in sub-Saharan Africa whose population of about 19 million people is among the poorest in the world. Eighty per cent of the rural population lives under the poverty line of US$1.90 (World Bank, 2018b). The population is frequently exposed to catastrophic shocks such as droughts and food supply disruptions, and lives in a high health risk environment, characterized by a low life-expectancies at birth of 59 years and a high HIV prevalence of 10% amongst adults (age 16–49) (World Health Organization, 2015). All of these factors have been found to be negatively associated with good mental health, creating a particularly high-risk mental health environment and posing a high threat for individual economic development (Petrushkin *et al.*, 2005; Antelman *et al.*, 2007; Rabkin, 2008; Lund *et al.*, 2010, 2011; Catalan *et al.*, 2011; Hanandita and Tampubolon, 2014; Angeles *et al.*, 2019; Kilburn *et al.*, 2019).

### Malawian longitudinal study of family and health

We use the 2006 and 2008 waves of the MLSFH, a longitudinal study of adults (age 16+) living in three rural districts in central (Mchinji), southern (Balaka) and northern (Rumphi) Malawi. The participants were randomized across 145 villages from the three regions. The sample of individuals followed-up across waves is representative of the rural adult population and contains information on socio-economic status, household characteristics, economic shocks, health outcomes, HIV status and health behaviours (Kohler *et al.*, 2015).

### Malawi incentive programme

**Figure 1:**
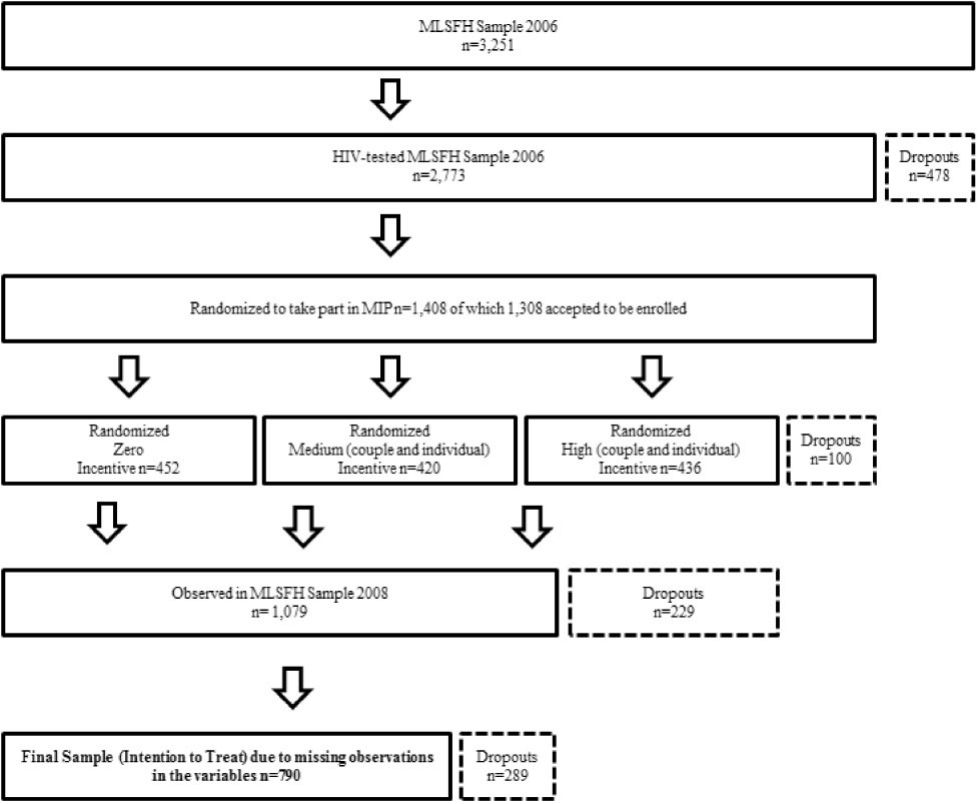
Composition of the estimation sample of the analysis.

Individuals enrolled in the 2006 MLSFH survey round were offered free HIV-tests and counselling on HIV-testing, as well as on HIV-risk factors and health effects. About 92% of the 3251 individuals accepted the test. Of the tested individuals, 1402 (43%) individuals were randomized for participation into the MIP, either with or without their partner, covering 145 different villages with treated and untreated individuals living in the same villages. Both HIV positive and negative individuals were included to avoid stigmatization. Figure 1 illustrates the sample composition.

A lump sum was transferred to randomly selected individuals or couples among those who would maintain their HIV status for at least 1 year (Kohler and Thornton, 2012). Of the 1402 selected individuals, 1308 (93%) individuals enrolled in the MIP and were then randomized into three groups: (1) untreated, (2) treated with the smaller cash transfer (Malawian Kwacha MKW 500 per individual/MKW 1000 per couple); or (3) treated with the larger cash transfer [MKW (Malawian Kwacha 2000 per individual or MKW 4000 per couple)]. The amounts of MKW 1000 or MKW 4000 were offered to couples jointly maintaining their HIV status. The magnitude of these cash offers was significant, as the average daily income in rural areas in Malawi amounts to MKW 20 (US$0.2) for men and MKW 10 (US$0.1) for women in 2006.

Individuals were visited four times during the trial. During the third round of interviews in 2007, about a year after the intervention had started, a second HIV-test was then made to verify the maintenance of the HIV status, which the cash transfer was tied to. Only after the third round, cash was paid out to couples or individuals that had maintained their HIV status. Attrition amongst treated and untreated in the cash transfer programme was relatively low, with only 142 dropouts (10%). Unlike previous studies, we focus on individual mental health outcomes. We combine the two treated groups to assess the effect of the programme on the individual level. The same approach was applied in the primary analysis of MIP programme effects on HIV incidence (Kohler and Thornton, 2012).

A possible concern for the identification of treatment effects of the cash transfer on mental health is that the MIP was targeting HIV-outcomes. Changes in mental health could be driven by HIV status rather than the cash transfer itself. However, previous analysis of the MIP has found no significant effects of the programme on HIV incidence (Kohler and Thornton, 2012). Therefore, treated and untreated are expected not to differ in their HIV status. This is important as otherwise changes in HIV status induced by the cash transfer programme could likely affect the respondent’s mental health which could bias the identification of the causal effects of the cash transfer on mental health. However, we also test if pre- and post-transfer HIV status affects our findings .

### Variables and descriptive statistics

#### SF12 mental health measure

We use the SF12 mental health scale (SF12), which is a good measure of general mental health (Macran *et al.*, 2003), and a reliable measure of mental health over time (Jenkinson *et al.*, 1997; Vilagut *et al.*, 2013). Previous research showed that the SF12 is a good screening tool for mild to moderate common mental health disorders such as depression, anxiety and psychological distress but may not be suitable for severe mental illnesses such as schizophrenia or psychosis (Vilagut *et al.*, 2013; Ohrnberger *et al.*, 2020). The SF12 consists of 12 questions related to physical health and to mental health (Ware *et al.*, 1995). To compute the respective health dimension of the SF12, weights or factor loadings are derived from principal component analysis. We use derived weights and validated the SF12 mental health dimensions for the Malawian population which is published elsewhere (Ohrnberger *et al.*, 2020). The SF12 has a maximum value of 100, indicating best possible mental health, and a minimum value of 0. The range of the SF12 makes it ideal for heterogenous effect analysis.

#### Control variables

We use a set of control variables to test the balance between treated and untreated groups and to improve precision in the estimation (Wooldridge, 2001). As social interactions are a strong determinant of mental health (Bekele *et al.*, 2013), we control for a set of binary variables indicating the respondent’s level of social integration and frequency of social interaction. We include variables indicating if the individual is a member of a local AIDS committee and how often in the past month the individual has been to a place to see a drama, to dance, to drink beer and/or to the market.

We use measures of self-perceived environmental risk to account for environmental risk factors that are associated with worse mental health outcomes (Turley *et al.*, 2013). These measures are individual perceived AIDS prevalence in community, the probability of infant mortality, the probability of a drought or equivalent food shock in the next 12 months, the number of people who have died as a result of AIDS known by the respondent and the respondent’s number of funeral visits in the past month.

We measure health behaviours by alcohol consumption and by smoking status, which have been shown to be strongly negatively associated with mental health outcomes (Whiteford *et al.*, 2013). We use a binary variable indicating if the individual ever smoked, one if he/she is currently smoking and one measuring the average number of days a week alcoholic drinks are consumed. Smoking and alcohol measures are frequency based and could mis-represent the actual consumed amount of alcohol or smoked cigarettes (Berggren and Sutton, 1999). For example, some individuals may drink few units of alcohol per day but on average more often compared with other individuals consuming alcohol less frequently but significantly more units of alcohol when they are drinking. However, the frequency of smoking cigarettes and drinking alcohol can still give an indication of consumption levels and substance abuse which is a common cause of mental health issues in LMICs (Lund *et al.*, 2011).

Good mental health is also associated with higher income and wealth (Golberstein and Busch, 2014). Direct measures of income and wealth are difficult to quantify as remunerating activities are often carried out in the informal sector and the measures of earnings rely on subjective recalling and reporting. We use instead a binary variable indicating if the individual lives in a house with a metal roof as a proxy for income/household wealth (Kohler *et al.*, 2015). This binary measure is commonly used in studies on the MLSFH to capture variations in income and wealth (Chin, 2010; Baranov *et al.*, 2015; Kohler *et al.*, 2015).

Previous research has found a strong correlation of better mental health with negative HIV status and well-being and vice-versa (Petrushkin *et al.*, 2005). We include a measure of subjective well-being and a binary variable indicating the HIV status of the individual.

In addition to this, we control for ethnic background (Yao, Tumbuka, Chewa or other ethnicity), which has varying impacts on mental health in the Malawian population (Kalembo *et al.*, 2019), educational attainment (none, primary, secondary tertiary), marital status (binary variable), the number of children living in the household, age and gender, and the number of regular household members. We include a set of dummy variables for region of origin (North, South, Central) to account for regional variations impacting mental health such as varying such as varying environmental conditions and access to (mental) health care (Kauye and Mafuta, 2007), and an indicator of whether the respondent was participating in the trial as an individual or in a couple incentive. All control variables are measured at baseline.

#### Descriptive statistics

Table 1 provides a comparison of the main variables between the treated and untreated participants. Mean mental health at baseline in 2006 is 50.2 in the untreated group and 49.6 in the treated group. In comparison, post-intervention mental health in 2008 changes in the treated group to 50.9 and in the untreated group to 49.9. About 60% of the samples are females, 94% are married and education is low on average, ranging between none and primary education. The samples are balanced by ethnic group and region. HIV prevalence varies from 7.5% in the treated group to 8.2% in the untreated group. 12.5% in the treated sample live in a house with a metal roof compared with 9% in the control sample. Fifteen per cent of the untreated and 18% of the treated currently smoke and individuals drink alcohol on average less than once a week.

**Table 1:** Descriptive statistics and test of balance of means of treated and untreated at baseline

Variable	Description	Untreated mean (SD)	Treated mean (SD)	*P*-values
Mental health	SF12 Mental health scale, 0 worst and 100 best mental health	50.2 (10.6)	49.6 (9.8)	0.421
Individual cash	1 if the individual cash, 0 if couple cash.	0.746	0.720	0.437
Female	1 if female, 0 if male	0.623	0.594	0.426
Age	Age in years	**35.9 (11.5)**	**39.0 (12.6)**	**0.001**
Education	0 ‘No school’, 1 ‘Primary’, 2 ‘Secondary’, 3 ‘Higher’	0.8 (0.6)	0.778	0.468
Other ethnicity	Other ethnic background	0.168	0.153	0.594
Yao	Yao ethnicity	0.306	0.291	0.667
Chewa	Chewa ethnicity	0.265	0.259	0.849
Tumbuka	Tumbuka ethnicity	0.261	0.297	0.293
Central	Central region	0.287	0.266	0.531
South	Southern region	0.433	0.408	0.504
North	Northern region	0.280	0.326	0.188
Married	1 if married 0 otherwise	0.944	0.935	0.614
HIV	1 if HIV positive 0 otherwise (VCT-counsellor tested)	0.082	0.075	0.714
Metal roof	1 if the house has a metal roof, 0 otherwise	0.090	0.128	0.106
Children	Number of children living in the household	**4.0 (2.6)**	**4.5 (3.0)**	**0.024**
Household size	Number of regular household members	**11.6 (3.6)**	**12.3 (4.3)**	**0.022**
Smoking	1 if smokes, 0 otherwise	0.149	0.178	0.305
Ever smoked	1 if ever smoked, 0 otherwise	0.205	0.234	0.364
Alcohol	Average number of days a week alcoholic drinks are consumed	0.4 (0.8)	0.4 (0.8)	0.799
AIDS committee	1 if member of the local AIDS committee, 0 otherwise	0.082	0.094	0.584
Funeral	Times individual has been to a funeral in past month	3.4 (2.5)	3.4 (2.2)	0.826
Drama	Times individual visited a drama place in past month	0.8 (1.9)	0.7 (1.6)	0.306
Dance place	Times individual visited a dance place in the past month	0.3 (1.1)	0.1 (0.7)	0.095
Beer place	Times individual visited a beer-drinking place in past month	1.0 (3.9)	0.8 (3.2)	0.513
Market	Times individual visited the market in past month	6.2 (6.4)	5.9 (5.7)	0.543
AIDS died	Number of individuals known to have died of AIDS	7.7 (7.2)	8.9 (8.7)	0.055
Prevalence AIDS	Self-ranked AIDS prevalence with 0 none and 10 very high	2.9 (1.7)	2.9 (1.6)	0.626
Infant mortality	Likelihood of infant mortality within 1 year after birth	**0.24**	**0.27**	**0.047**
Food shortage	Likelihood of food shortage within 1 year	0.52	49.83	0.333
Well-being	Subj. well-being from 0 ‘Very unsatisfied’ to 4 ‘Very satisfied’	3.1 (1.0)	3.0 (1.0)	0.397

*T*-test of means comparing the treated (522) with untreated (268). Variable means with significant differences are in bold (*P* < 0.05).

Individuals attend a funeral about three times in the 30 days before the interview. Individuals visited on average a place to see a drama and a place to drink a beer once a month, and a place to dance and the market six times a month. The average number of people known by the individual to have died of AIDS ranges between nine (treated) and eight (untreated). The respondent expects every third person living in the same area to have AIDS. The individually perceived chance of food shortage within a year is 50%. The comparison of the means of the treated and untreated participants shows that the randomization produced comparable groups and likewise for the comparison of means by treatment group and within quintiles of the SF12 mental health distribution (Table 2).

**Table 2:** Test of mean differences between treated and untreated by quantiles at baseline

	10th quantile	25th quantile	50th quantile	75th Quantile	90th Quantile
	*P*-value	*P*-value	*P*-value	*P*-value	*P*-value
MH Malawi	**0.026**	0.581	0.994	0.139	0.134
Individual cash	0.568	**0.029**	0.230	0.133	0.693
Female	0.980	0.901	0.567	0.835	0.106
Age	0.855	0.342	**0.014**	0.414	**0.015**
Education	0.113	0.766	0.574	0.290	0.851
Other ethnicity	0.574	0.121	0.978	0.315	0.763
Yao	0.401	0.391	0.831	0.264	0.897
Chewa	0.491	0.552	0.463	0.629	0.299
Tumbuka	0.374	0.280	0.424	0.924	**0.038**
Central	0.796	0.261	0.839	0.458	0.363
South	0.349	0.856	0.676	0.682	0.402
North	0.374	0.214	0.576	0.810	**0.011**
Married	0.838	0.584	0.726	0.107	0.105
HIV	0.881	0.660	0.311	0.426	0.293
Metal roof	0.051	0.621	0.304	0.619	0.519
Children	0.750	**0.016**	0.120	0.859	0.103
Household size	0.551	0.423	**0.035**	0.557	0.104
Smoking	0.704	0.418	0.312	0.360	0.214
Ever smoked	0.598	0.391	0.171	0.543	0.352
Alcohol	0.572	0.341	0.557	0.461	0.366
AIDS committee	0.257	0.240	0.508	0.208	0.753
Funeral	0.875	0.377	0.852	0.377	0.449
Drama	0.449	0.312	**0.033**	0.416	0.453
Dance place	0.481	0.240	0.147	0.713	0.471
Beer place	0.392	0.152	0.279	0.104	0.171
Market	0.678	0.264	0.374	0.379	0.918
AIDS died	0.765	0.572	0.342	0.634	**0.033**
Prevalence AIDS	0.649	**0.012**	0.598	0.767	0.555
Infant mortality	0.922	0.680	0.295	0.165	0.250
Food shortage	0.132	0.843	0.882	0.631	0.434
Well-being	0.893	0.800	0.717	0.793	0.543

*P*-values of *t*-test of means comparing the treated quantiles with the respective untreated quantiles at baseline; 10th Quantile: 55 Untreated (U) vs 112 Treated (T); 25th Quantile: 49 U vs 112; 50th Quantile: 61 U vs 114 T; 75th Quantile: 45 U vs 93 T; 90th Quantile: 58 U vs 91 T. Variable means with significant differences are in bold (*P* < 0.05).

Whilst attrition in the cash transfer programmes was relatively low, about 10%, attrition in our estimation sample is about 40%. Attrition is larger in the estimation sample due to attrition of cash transfer participants in the post-intervention round of the MLSFH in 2008 and missing observations for some participants in the mental health measure post-intervention. Table 3 shows a good balance in characteristics between the estimation sample and the attritors, with difference in some individual and socio-economic characteristics such as gender, age or education.

**Table 3:** Mean comparison of characteristics between estimation sample and attritors

	Attrition, mean (SD)	Estimation sample, mean (SD)	*P*-value
MH Malawi	49.1	49.8	0.314
Individual cash	**0.813**	**0.729**	**0.000**
Female	**0.503**	**0.604**	**0.000**
Age	**32.4 (13.9)**	**38.0 (12.3)**	**0.000**
Education	**1.0 (0.7)**	**0.8 (0.6)**	**0.000**
Other ethnicity	0.143	0.158	0.425
Yao	0.292	0.296	0.867
Chewa	0.277	0.261	0.508
Tumbuka	0.289	0.285	0.877
Central	0.293	0.273	0.414
South	0.398	0.416	0.489
North	0.309	0.310	0.951
Married	**0.648**	**0.938**	**0.000**
HIV	**0.116**	**0.077**	**0.014**
Metal roof	0.152	0.115	0.053
Children	**3.0 (3.6)**	**4.3 (2.9)**	**0.000**
Regular household members	**10.3 (4.6)**	**12.1 (4.1)**	**0.000**
Smoking	0.199	0.168	0.203
Ever smoked	0.270	0.224	0.094
Alcohol	0.4 (0.8)	0.4 (0.8)	0.288
AIDS committee	0.128	0.090	0.050
Funeral	3.3 (2.3)	3.4 (2.3)	0.734
Drama	0.8 (2.1)	0.7 (1.7)	0.694
Dance place	0.2 (0.8)	0.2 (0.9)	0.939
Beer place	0.9 (3.2)	0.9 (3.4)	0.870
Market	6.6 (7.1)	6.0 (5.9)	0.145
AIDS died	8.1 (8.2)	8.5 (8.2)	0.407
Prevalence AIDS	2.9 (1.8)	2.9 (1.6)	0.678
Infant mortality	2.6 (2.2)	2.6 (2.2)	0.759
Food shortage	5.0 (3.1)	5.1 (3.0)	0.617
Well-being	3.0 (1.0)	3.0 (1.0)	0.376

Comparison of means of the estimation sample (790 individuals) with the attrition sample (593) which is composed of all individuals invited to take part in the CCT but rejected (75), individuals that took part in the CCT but dropped out during the CCT and are not observed in the 2008 MLSFH survey round (102; 49 of the 102 were offered 0-cash), and with individuals that took part in the CCT and remained in the CCT, but are either not observed in the 2006 MLSFH survey round and/or not observed in the 2008 MLSFH survey round (survey attrition = 416). Variable means with significant differences are in bold (*P* < 0.05).

## Methods

### Analysis of average and quantile treatment effects

We estimate the following model:

(1) $$y_{i}=\beta_{0}+\beta_{1}D_{i}+\beta_{2}y_{i,t=0}+X_{i,t=0}\beta_{3}+\epsilon_{i}$$

where $y_{i}$ is mental health before and after the intervention, for individual *i*. $D_{i}$ is a binary variable taking the value of one for treated and zero for untreated individuals. $\beta_{1}\;$is the coefficient of interest measuring the average effect of the MIP. To increase precision, we control for the full set of covariates at baseline represented by the vector $X_{i,\;t\;=\;0}$ and include baseline mental health $y_{i,\;t\;=\;0}$ in the estimation which is a common approach in the literature (Wooldridge, 2001; Baird *et al.*, 2013).

To understand treatment heterogeneity across the distribution of mental health, we estimate the QTE in equation (2),

(2) $$y_{i,\;t=1}=Q_{y^{1}}^{\tau }-Q_{y^{0}}^{\tau }|\;X_{i,\;t=0},\;y_{i,\;t=0}$$

where $Q_{y^{1}}^{\tau }-Q_{y^{0}}^{\tau }$ is the QTE at quantile $\tau \;\epsilon \;\left(0.1,\;0.25,\;0.5,\;0.75,\;0.9\right)$ derived by taking the difference between the τ quantile of the mental health distribution for treated $Q_{y^{1}}^{\tau }$ and untreated $Q_{y^{0}}^{\tau }$. The outcome measure $y_{i,\;t\;=\;1}$ is mental health and $X_{i,t=0}$ are the covariates at baseline. The coefficient estimates from the QTE are interpreted conditional on the estimated quantile, which is like the interpretation of OLS coefficients conditional on the mean.

We bootstrap the standard errors to retain the assumption of independent errors and relax the assumption of identically distributed errors and obtain robust standard errors. The interpretation of distributional effects of the CCT on mental health to individual treatment effects requires the assumption of rank preservation, which is that the relative rank of an individual in the outcome distribution is the same with and without treatment (Frandsen and Lefgren, 2018). We test this assumption and prefacing the findings we find strong support for the assumption to hold.

### Treatment effect interactions with baseline HIV and gender

We test for heterogeneous effects by HIV status at baseline because maintaining the HIV status is the conditionality rule attached to the cash transfer. We test for heterogeneous effects by gender as a previous study found mixed evidence for heterogeneity by gender of CCT effects on child mental health outcomes in Mexico, with no heterogeneity in depression and anxiety and heterogeneity in aggressive symptoms (Ozer *et al.*, 2009). We follow Ozer *et al.* (2009) and estimate interaction effects. We re-estimate equations (1) and (2) and interact treatment with HIV status and binary variables for gender.

### Controlling for effects on post-intervention HIV status

We revisit equations (1) and (2) and include post-intervention HIV status in the estimation. Changes in the post-intervention HIV status can negatively affect mental health for having contracted HIV, for both treated and untreated individuals. In addition, since the cash transfer receipt was tied to remaining in one’s HIV status, the loss of the potential cash transfer may have negative effects on the mental health of HIV-status switchers among the treated. No significance in the effect of post-intervention HIV status would support the claim that effects of the cash transfer truly effect mental health.

### Usage of the cash transfer and mental health

The usage of the cash transfer can affect mental health differently. Following the Grossman model of health in which health is considered an investment good, the additional income from the cash transfer could be used to purchase more nutritious food with consequent mental health improvements (Grossman, 1972; Ohrnberger *et al.*, 2017). The social causation hypothesis of mental health disorders suggests that more disposable income could also reduce pressure and stress to provide necessary support for the family (Lund *et al.*, 2011; Lund and Cois, 2018). Investments into private business can translate into more planning security or more productivity that can reduce financial anxiety and improve mental health.

To understand how cash transfer may affect mental health, we use information about how the cash transfer recipients used the transfer. We re-estimate equations (1) and (2) for the sample of cash-transfer recipients and include variables indicating the usage of the cash. We use the following variables reflecting expenditure as explanatory variables: first, expenditure related to productivity (96 of 476 individuals), such as buying fertilizer, hiring labour or buying seeds; second, expenditures related to consumption (373 of 476 individuals), such as household goods, food, or clothes and textiles; third, expenditures related to child education (19 of 476 individuals), such as tuition fees or textbooks; fourth, expenditures related to transport (31 of 476 individuals), such as bicycle taxis; fifth, expenditures related to health (12 of 476 individuals), such as medicine, cost of a local healer or the fee for the doctor, and sixth other expenditures (68 of 476 individuals).

### Testing the rank preservations (invariance) assumption

Rank invariance is required in QTE models to identify casual individual treatment effects. If the assumption does not hold, effects are interpreted as distributional but not individual effects (Angrist and Pischke, 2008; Frandsen and Lefgren, 2018). Chernozhukov and Hansen (2005) introduced the concept of rank preservation assumption which requires the conditional distribution of the ranks, and not the ranks of the individual, to be identical in treatment states. Rank preservation does not exclude rank invariance. We use two approaches to test for rank preservation.

Firstly, we identify the ratios of mental health quantile switchers from pre-intervention to post-intervention for both groups of treated and untreated. As we are using an RCT and the comparison of means by treatment status and quantiles has shown a strong balance in characteristics between the groups, we can assume that individuals also have good counterfactuals by quantiles and not just on average. Using this assumption, we can identify the quantile switchers in untreated and treated groups and can relate them to each other.

Secondly, we follow the methodology of Frandsen and Lefgren (2018). Their rank test compares the distribution of treated and untreated conditional on a rank-shifting variable *S*. Rank preservation holds if the distributions of treated and untreated conditional on *S* are identical. The rank-shifting variable needs to be correlated with the outcome in the absence of treatment, uncorrelated with treatment status and observed at baseline. We define *S* as a binary variable indicating if an individual had above-average mental health at baseline, so that it satisfies the properties of a rank-shifting variable. Individuals with better mental health should also have better mental health after the intervention, irrespective of treatment status. Mental health is equally distributed among treatment groups at the onset of the study indicating independence of treatment status with mental health.

To test for rank preservation, we compute the post-intervention mental health rank within treatment status using the cumulative distribution function to identify the rank of the individual. Firstly, we graphically show: (1) how the rank distribution of post-intervention mental health within each treatment state behaves conditional on the rank-shifting binary variable for the group of untreated; (2) how it is distributed among treated and untreated below-average health; and (3) the distribution of *S* among the group of individuals above-average mental health by treatment status. Secondly, we estimate equation (3):

(3) $$\hat{U_{i}}=\;\beta_{0}+\;\beta_{1}D_{i}+{\beta_{2}S}_{i}+{\beta_{3}D}_{i}\times S_{i}+\;\epsilon_{i}$$

where $\hat{U_{i}}$ is the individual sample rank in post-intervention mental health conditional on treatment status, $D_{i}$ is a binary variable taking the value of 1 if the individual received the treatment and 0 otherwise. $S_{i}$ is a binary variable taking the value of 1 if an individual has above-average mental health at baseline 0 otherwise, and $\beta_{2}$ establishes the power of the rank-shifting variable. $D_{i}\times S_{i}$ is the interaction of treatment status with the rank-shifting variable. We use the $\beta_{3}\;$associated with the interaction term to test the independence of treatment from *S*, i.e. to test the null hypothesis for rank preservation (Frandsen and Lefgren, 2018).

## Results

### Analysis of average and quantile treatment effects

**Figure 2:**
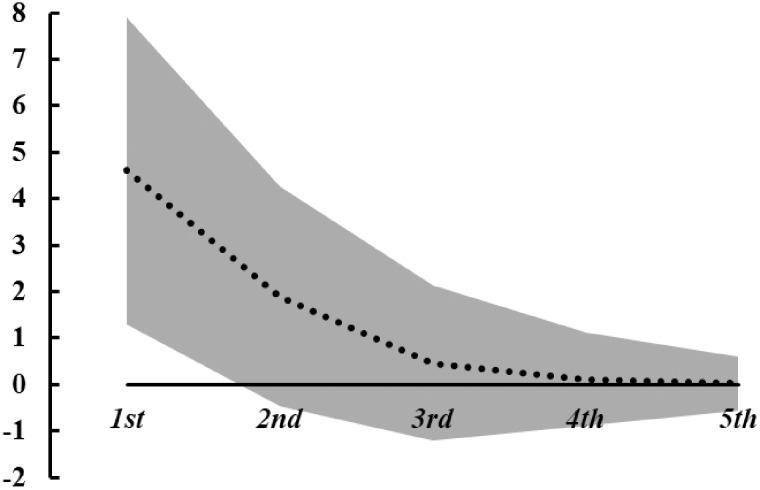
QTEs on mental health with 95% CI band. The outcome is mental health, 1st to 5th refer to the estimated quantiles (Q0.1–Q0.9). The dotted line is the estimate quantile effect. The grey area is the 95% CI of the estimated quantile effects.

We present in Table 4 and Figure 2, the results of the quantile treatment effect by quantiles of mental health along with the average treatment effect on mental health. The results for the lowest quantile in column (1) show a positive and significant and with size of 4.6, which is about half of a standard deviation in mental health. The treatment effect is only significant in the lowest quantile. The magnitude of the effect decreases from quantile one to five. The average effect in column (6) is positive and significant with size 1.1. The QTE for the lowest quantile is about four times as high as the average effect. The dotted line in Figure 2 illustrates the downward sloping QTEs from worst to poor mental health quantiles. The grey area around the dotted line is the 95% confidence interval of the estimated QTEs.

**Table 4:** QTE regression on mental health

	(1)	(2)	(3)	(4)	(5)	(6)
	Q(0.1)	Q(0.25)	Q(0.50)	Q(0.75)	Q(0.9)	Average Effect
						
Treated	4.599^***^	1.900	0.458	0.116	0.021	1.124^*^
	(1.690)	(1.200)	(0.852)	(0.512)	(0.296)	(0.640)
MH baseline	0.334^***^	0.261^***^	0.276^***^	0.155^***^	0.040	0.216^***^
	(0.116)	(0.078)	(0.060)	(0.033)	(0.034)	(0.049)
Constant	12.347	35.025^***^	43.526^***^	53.298^***^	59.915^***^	42.259^***^
	(9.984)	(7.213)	(4.402)	(2.657)	(2.289)	(3.812)
						
Baseline covariates	Yes	Yes	Yes	Yes	Yes	Yes

The outcome variable is mental health by quantiles after the intervention for (1) to (5). The outcome variable in (6) is the continuous mental health variable. The sample size is 790. Bootstrapped standard errors for quantiles are in parenthesis; robust standard errors for the average effect are in parenthesis. We bootstrapped the estimates on 500 repetitions.

*** *P* < 0.01,

** *P* < 0.05,

* *P* < 0.1.

### Treatment effect interactions with baseline HIV and gender

Table 5 presents the findings from the QTE columns (1) to (5) and average effect analysis in column (6) using baseline interactions of treatment and HIV status or gender in model (1). Results from the first model do not show a significant effect for the interaction of HIV status with treatment for both QTE and average effect, indicating that the effect of CCT on mental health is not different between the treated HIV positive and HIV negative. The treatment effect for the HIV negative is positive and significant only in the lowest mental health quantile with effect size of about half an SD (4.5) in mental health. The effect size decreases with increasing mental health quantiles.

**Table 5:** Average effect and QTE-interaction models of HIV at baseline and gender with treatment

	(1)	(2)	(3)	(4)	(5)	(6)
	Q(0.1)	Q(0.25)	Q(0.50)	Q(0.75)	Q(0.90)	Average effect
Model (1) Estimation of QTE by HIV status at baseline						
Treated	4.486^***^	1.904	0.458	0.215	0.054	1.056
	(1.718)	(1.271)	(0.866)	(0.491)	(0.326)	(0.638)
HIV baseline	−1.573	0.073	−1.009	−0.944	−0.325	−1.993
	(9.200)	(3.728)	(3.280)	(2.188)	(1.320)	(2.523)
Interacted treated with HIV baseline	0.757	0.347	−0.594	−0.696	−0.339	0.874
	(10.546)	(4.196)	(3.732)	(2.409)	(1.864)	(2.864)
MH baseline	0.338^***^	0.258^***^	0.276^***^	0.156^***^	0.037	−0.784^***^
	(0.116)	(0.080)	(0.060)	(0.035)	(0.035)	(0.049)
Constant	11.748	35.544^***^	43.526^***^	53.165^***^	59.941^***^	42.255^***^
	(9.814)	(7.069)	(4.254)	(2.625)	(2.485)	(3.818)
Baseline covariates	Yes	Yes	Yes	Yes	Yes	Yes
Model (2) Estimation of QTE by gender						
Treated	3.948^*^	3.128	0.877	0.643	0.296	−1.376
	(2.333)	(1.966)	(1.442)	(0.749)	(0.522)	(1.299)
Female	−3.338	−1.515	−1.830	−0.548	−0.949	−1.533
	(2.961)	(2.215)	(1.707)	(0.845)	(0.628)	(1.338)
Interacted treated with female	1.802	−2.249	−0.639	−0.826	−0.428	−0.710
	(3.353)	(2.505)	(1.877)	(0.975)	(0.653)	(1.338)
MH baseline	12.022	35.974^***^	43.046^***^	52.864^***^	58.738^***^	−0.785^***^
	(9.479)	(6.549)	(4.588)	(2.602)	(2.302)	(0.049)
Constant	0.357^***^	0.239^***^	0.279^***^	0.158^***^	0.046	42.061^***^
	(0.111)	(0.072)	(0.063)	(0.033)	(0.032)	(3.781)
Baseline covariates	Yes	Yes	Yes	Yes	Yes	Yes

The outcome variable is mental health by quantiles after the intervention for (1) to (5). The outcome variable in (6) is the continuous mental health variable. The sample size is 790. Bootstrapped standard errors are in parenthesis. We bootstrapped the estimates on 500 repetitions. We estimate in Model (1), the interaction of treatment with HIV at baseline. Model (2) estimates the treatment effect interacted with gender.

*** *P* < 0.01,

** *P* < 0.05,

* *P* < 0.1.

Model (2) includes the interaction of gender with treatment. We do not find a significant effect of the interaction of female with treatment status for both QTE and average effect, suggesting no statistically significant difference in treatment effect by gender. The treatment effect for male is positive and only significant in the first quantile of mental health and of size 3.9. As for the model including the interaction between treatment and HIV status, the quantile effect size decreases with increasing quantiles.

### Controlling for effects on post-intervention HIV status

Table 6 presents the estimation results after including post-intervention HIV status. Post-intervention HIV status is not significant in predicting mental health in either QTE or average effect estimation. The estimated QTE and average effects are robust to inclusion of the post-intervention HIV status which suggests that the focus of the cash transfer namely to retain one’s HIV status does not affect or bias the estimations of the CCT on mental health.

**Table 6:** Controlling for post-intervention HIV status in average effect and QTE estimation

	(1)	(2)	(3)	(4)	(5)	(6)
	Q(0.1)	Q(0.25)	Q(0.50)	Q(0.75)	Q(0.9)	Average effect
Treated	4.704^***^	1.826	0.624	0.129	−0.064	1.162^*^
	(1.764)	(1.254)	(0.979)	(0.544)	(0.331)	(0.627)
Post-HIV	−5.229	3.068	−4.504	2.088	0.343	−2.283
	(8.540)	(8.378)	(6.629)	(5.439)	(4.135)	(5.328)
Constant	14.762	36.969^***^	43.300^***^	51.314^***^	56.029^***^	41.250^***^
	(10.466)	(7.082)	(4.519)	(2.735)	(2.186)	(4.218)
Baseline covariates	Yes	Yes	Yes	Yes	Yes	Yes

The outcome variable is mental health by quantiles after the intervention for (1) to (5). The outcome variable in (6) is the continuous mental health variable. The sample size is reduced to 734 for missing observations in the post-intervention HIV variables. Bootstrapped standard errors for quantiles are in parenthesis; clustered standard errors for the average effect are in parenthesis. We bootstrapped the estimates on 500 repetitions.

*** *P* < 0.01,

** *P* < 0.05,

* *P* < 0.1.

### Usage of the cash transfer and mental health

Columns (1) to (5) of Table 7 present the findings for the QTE analysis on post-transfer mental health and column (6) presents the average effect on mental health. The sample size for the cash transfer recipients is reduced from 522 to 476 observations due to missing information on cash expenditure. QTE on the treated are statistically significant for the lowest quantile of mental health in model (1) and for the average effect in model (6). Looking at model (2), we find significant positive associations with using cash for productivity- and consumption-related expenditures in the lowest two quantile and on average. At the median (q0.5), positive changes in mental health are related to productivity expenditures. The findings represent only associations but suggest that the cash transfers may affect mental health positively due to increased consumption- and productivity-related expenditure.

**Table 7:** Associations of usage of money on average changes in mental and quantile changes in mental health

	(1)	(2)	(3)	(4)	(5)	(6)
	Q(0.1)	Q(0.25)	Q(0.5)	Q(0.75)	Q(0.9)	Average Effect
Cash for	8.012^***^	6.035^**^	3.226^*^	1.115	0.876	3.394^**^
productivity	(2.808)	(2.446)	(1.643)	(1.068)	(1.141)	(1.318)
Cash for	7.662^***^	6.771^***^	2.604	0.614	0.809	3.119^**^
Consumption	(2.872)	(2.577)	(1.936)	(1.193)	(1.138)	(1.256)
Cash for education	4.247	−1.735	−1.737	−1.049	0.270	−0.398
	(4.273)	(3.899)	(3.191)	(2.569)	(2.236)	(1.609)
Cash for transport	5.553	2.180	0.518	−0.171	0.493	0.539
	(3.605)	(3.047)	(2.165)	(1.400)	(1.083)	(1.528)
Cash for health	1.278	−4.838	−0.884	−2.498	−1.953	−3.359
	(5.457)	(4.814)	(5.074)	(2.968)	(1.810)	(2.833)
Cash for other	−0.527	0.489	0.758	0.523	0.258	−0.075
	(3.280)	(2.196)	(1.433)	(1.028)	(0.739)	(1.260)
MH baseline	0.492^***^	0.292^***^	0.340^***^	0.136^***^	0.076^*^	−0.717^***^
	(0.113)	(0.091)	(0.078)	(0.052)	(0.046)	(0.056)
Constant	6.736	27.226^***^	37.694^***^	50.420^***^	55.621^***^	34.184^***^
	(11.553)	(8.824)	(6.022)	(4.196)	(3.599)	(4.870)
Baseline covariates	Yes	Yes	Yes	Yes	Yes	Yes

The outcome variable is mental health by quantiles after the intervention for (1) to (5). The outcome variable in (6) is the continuous mental health variable. The sample size is reduced to 476, only transfer recipients with 46 missing observations. We estimate the Quantile Treatment Effect on the Treated (QTET) and the linear treatment effect for transfer recipients. Bootstrapped standard errors for quantiles are in parenthesis; robust standard errors in (6) are in parenthesis. We bootstrapped the estimates on 500 repetitions.

*** *P* < 0.01,

** *P* < 0.05,

* *P* < 0.1.

### Testing the rank preservation (invariance) assumption

**Figure 3:**
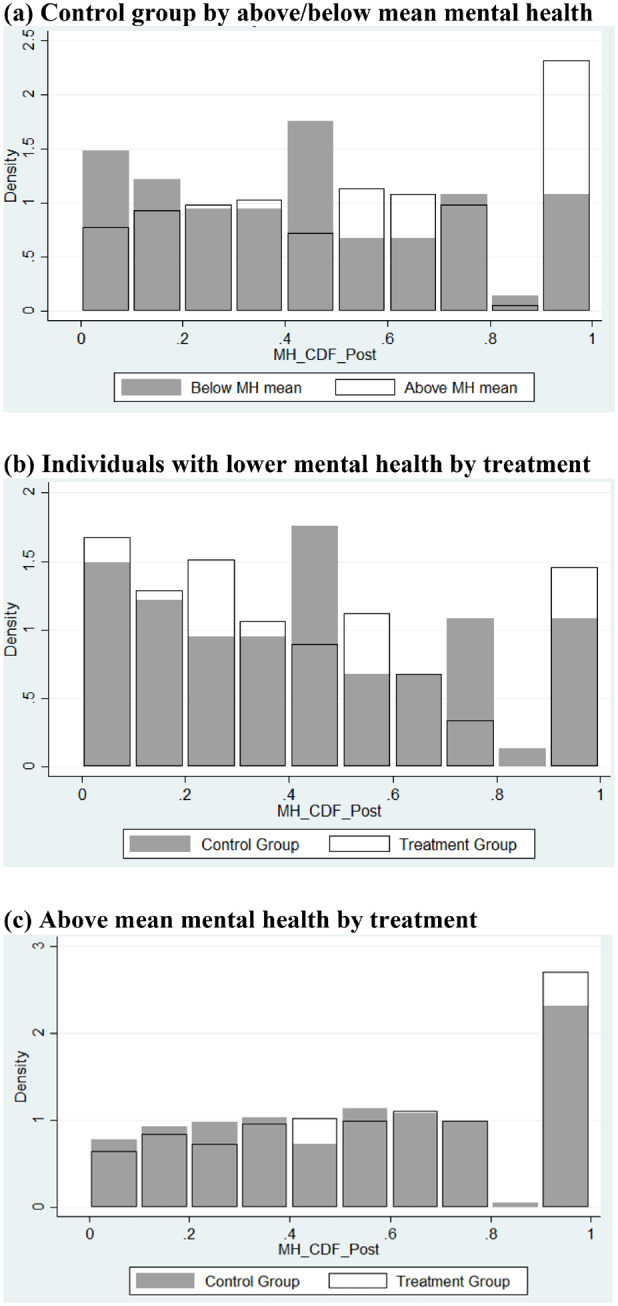
Rank distributions of mental health comparing specific baseline status to rank at post-intervention mental health.

We present two approaches to assess and test rank similarity and rank invariance. Table 8 presents the descriptive analysis and Table 9 with Figure 3 present the test following Frandsen and Lefgren (2018).

**Table 8:** Assessing the rank similarity (invariance) assumption: patterns in keeping and switching quantiles from pre- to post-intervention in treated and untreated

	Baseline 1st quantile	Baseline 2nd quantile	Baseline 3rd quantile	Baseline 4th quantile	Baseline 5th quantile
Untreated (U)					
Switch post-intervention quantile	32	38	48	39	51
No switch post-intervention quantile	14	11	13	15	7
Percentage switch	69.57	77.55	78.69	72.22	87.93
Treated (T)					
Switch post-intervention quantile	75	87	93	62	81
No switch post-intervention quantile	37	25	21	31	9
Percentage switch	66.96	77.68	81.58	66.67	90.11
Difference of percentages by Quantiles and Treatment Status					
Difference in percentages U v. T	2.61	−0.13	−2.89	5.55	−2.18

**Table 9:** Test of rank similarity (invariance) assumption for QTE

	(1)
	CDF MH post-intervention
Treated	−0.033
	(0.041)
High MH at baseline	0.100^**^
	(0.043)
Interacted treated with High MH at baseline	0.061
	(0.053)
Constant	0.442^***^
	(0.034)
Individuals	790
*R*-squared	0.049

The outcome is the rank of the individual on cumulative density distribution of post-intervention mental health within treatment status. Clustered standard errors in parentheses.

*** *P* < 0.01,

** *P* < 0.05,

* *P* < 0.01.

Table 8 gives the results of the rank similarity and invariance test. We find for the group of untreated that 69.57% switch from the pre-intervention first quantile, 77.55% from the second quantile, 78.69% from the third, 72.22% from the fourth quantile and 87.93% from the fifth quantile. We find similar percentages of switchers per quantiles amongst treated individuals (Q1: 66.96%; Q2: 77.68%; Q3: 81.58%; Q4: 66.67%; Q5: 90.11%). The differences between percentages of treated and untreated switchers by quantiles range between 0.13 (Q2) and 5.55 (Q4), suggesting that switching patterns are similar and irrespective of treatment status, which then can support the rank similarity (invariance) assumption.

Figure 3 shows the rank distribution of post-transfer mental health for individuals with mean and below mean baseline mental health and for individuals with above mean baseline mental health. We show in (a) how the rank distribution in the untreated group behaves with respect to whether individuals have above mean mental health or mean and below mean mental health at baseline. It is evident that the density of ranks is shifted to the right for higher levels of mental health at baseline compared with individuals with mean and below mean mental health at baseline. This suggests that the binary indicator satisfies the rank-shifting property.

In (b), we compare the distribution of ranks for both treated and untreated individuals, conditional on mean and below mean mental health at baseline. The middle part of the distribution has less mass in the treatment group compared with the control group. This pattern is similar for the top part of the distribution. Under the null hypothesis for rank similarity (invariance), the rank distributions should be similar. This suggests that rank similarity may not hold. In (c), we present the distribution of ranks for both treated and untreated individuals with above-average mental health at baseline. We find a similar distribution of mass for both groups.

Table 9 presents the statistical analysis of rank similarity. The rank-shifting variable is significant and taking on the value 0.1. This implies that those individuals in the control group with above-average mental health at baseline are located 10% higher in the outcome distribution than those individuals with average or below-average mental health at baseline. This supports, together with Figure 3, the strong power of the rank-shifting variable. The estimated effect of the interaction between the treatment and the rank-shifting variable has an effect of 0.061 but is not statistically significant. It implies that no rank disadvantage exists between treated and untreated and that the null hypothesis of rank similarity is not rejected. Our findings are to be interpreted as individual effects and not distributional effects.

## Discussion

This is the first study that estimates both the average and heterogeneous effects of a CCT programme on adult mental health in a low-income country for the adult population. We use an RCT sample of 790 adults from the MIP, a CCT with cash transfers receipt conditional on maintaining the HIV status for a year. We find that the average effect of the programme is significant and positive and shows an increase along the SF12 mental health measure of 1.1 units which is about 2% of the sample mean. We find heterogenous effects with improvements in mental health equal to 4.6 units for the lowest quantile of the mental health distribution. This effect is about four times the average effect. We find strong evidence in support of the rank preservation (invariance) assumption of the QTEs. Findings are thus interpreted as individual-level effects and not only as distributional effects.

We observe no significant treatment interaction effects with gender and baseline HIV status, neither in the average treatment effect analysis nor on the QTE analysis. Controlling for post-intervention HIV, the results remain robust. The cash transfer provides more capability to invest directly or indirectly in better mental health. Those individuals with worst mental health, benefit the most from the transfer. The positive associations of productivity- and consumption-related expenditures with the lowest mental health quantiles support this interpretation.

Our findings of positive average mental health effects among adults relate to previous analyses which found similar effects of CCTs in LMICs in population sub-samples of adolescents, mothers and children (Fernald and Gunnar, 2009; Ozer *et al.*, 2009, 2011; Baird *et al.*, 2013). Like in our analysis, these studies used programmes and incentives that were not aimed at improvements in mental health. The findings of strongest mental health effects for individuals with worst mental health are similar to those studies using other exogenous income variations, such as asset promotion or green-card lotteries for poor populations (Stillman *et al.*, 2009; Banerjee *et al.*, 2015).

The strong and positive treatment effect for the lowest quantiles of mental health can be interpreted using a human health capital model such as the Grossman model of health in which individuals aim to maximize their utility where health (here mental health) is both an investment and consumption good (Grossman, 1972). Accordingly, the cash transfer provides individuals with more capabilities to invest in their mental health. This links to the capabilities approach, according to which well-being improves because individuals are enabled to realize their capabilities (White *et al.*, 2016). Those individuals with the strongest need or least satisfied capabilities, e.g. those with the worst mental health, benefit the most from the transfer due to smaller marginal returns in health provided utility the higher one moves up the mental health distribution. Our findings of positive associations of productivity- and consumption-related expenditures of the cash transfer with the lowest mental health quantile support this interpretation.

Recent work by Lund and Cois (2018) identified a simultaneous relationship of social drift (worse mental health causes more poverty) and social causation (poverty causes worse mental health) of mental health problems in LMICs. CCTs can be a powerful tool to stop this vicious circle. As our findings show, providing additional income through a CCT improves mental health, which can stop the vicious circles by reducing the social drift. Improvements in mental health are associated with more capabilities to spend on consumption goods and productivity-related goods which can then improve the economic status and thus reduce the social causation problem.

A limitation of this study is the restriction to short-term effects of cash transfer programmes on mental health. One of the strengths of this study is that post-randomization selection or contamination of the controls with treatment group is very unlikely to happen due to the nature of the RCT and MLSFH. The design of the MLSFH permits all individuals that agreed on HIV-counselling a similar set of information regarding risk factors causing HIV. Learning effects should be similar for untreated and treated and should therefore not affect mental health differently in these groups. Another limitation is the high attrition rate among study participants. However, as characteristics between attritors and the estimation sample are mostly balanced, we do not expect our results to be affected by attrition bias.

The RCT was tailored to offer cash for maintaining HIV status. Potentially, untreated individuals living in proximity to treated participants might have positive spill-over effects due to the treated taking precautions in their sexual behaviour to remain in their HIV status. These spill-over effects are evident. However, they would not affect the outcome variable, as HIV status and environmental factors per se do not significantly affect the mental health measure. Therefore improvements in mental health are likely due to the exogenous financial shock and not due to remaining in the HIV status, as a previous study on the MIP has not found a significant effect of cash on individual HIV (Kohler and Thornton, 2012). Whilst the RCT was designed to analyse HIV effects, we test the power required for finding a 1-unit change in mental health using Satterthwaite’s *t*-test. The test shows that our sample size has enough power (1-β = 0.93) to detect such a change in mental health.

An ethical concern of the RCT design and the CCT is that the experience of windfall income may harm mental health post-intervention due to the sudden and discontinuous income gain. We cannot access such potentially detrimental effects in our study due to data limitations. An existing study by an existing study by Baird *et al.* (2011) of a randomized-controlled CCT in Malawi showed that no adverse effects on psychological distress occurred for either CCT-recipients or the control group in the time after the CCT.

## Conclusion

We have shown that CCT effects on mental health are significant and positive on average and strongest for individuals with worst mental health, without differences by gender. These cash transfers can increase individual capabilities to invest in productivity and consumption which are strongly related to improvements in mental health. Policymakers should consider cash transfers as a mean to improve adult mental health for the poor living in low-income settings, upon appropriate ethical considerations.
